# Quality of Life in European Older Adults of SHARE Wave 7: Comparing the Old and the Oldest-Old

**DOI:** 10.3390/jcm10132850

**Published:** 2021-06-27

**Authors:** Amparo Oliver, Trinidad Sentandreu-Mañó, José M. Tomás, Irene Fernández, Patricia Sancho

**Affiliations:** 1 Department of Methodology for the Behavioral Sciences, University of Valencia, 46010 Valencia, Spain; amparo.oliver@uv.es (A.O.); jose.m.tomas@uv.es (J.M.T.); irene.fernandez@uv.es (I.F.); 2 Department of Physiotherapy, University of Valencia, 46010 Valencia, Spain; 3 Department of Educational and Developmental Psychology, University of Valencia, 46010 Valencia, Spain; patricia.sancho@uv.es

**Keywords:** quality of life, older adults, oldest-old, measurement invariance, SHARE European Survey

## Abstract

CASP-12 (Control, Autonomy, Self-realization, and Pleasure scale) is one of the most common internationally used measures for quality of life in older adults, although its structure is not clearly established. Current research aims to test the factor structure of the CASP-12, so as to provide evidence on reliability and external validity, and to test for measurement invariance across age groups. Data from 61,355 Europeans (≥60 years old) from the Survey of Health, Ageing and Retirement in Europe wave 7 were used. CASP-12, EURO-D (European depression scale), self-perceived health, and life satisfaction measurements were included. Reliability and validity coefficients, competing confirmatory factor models, and standard measurement invariance routine were estimated. A second-order factor model with the original factor structure was retained. The scale showed adequate reliability coefficients except for the autonomy dimension. The correlation coefficients for external validity were all statistically significant. Finally, CASP-12 is scalar invariant across age. We conclude that the best-fitting factor structure retained allows using CASP-12 either by factors, or as an overall score, depending on the research interests. Findings related to CASP-12 measurement invariance encourage its use in the oldest-old too. When comparing the dimensions across age groups, as people age, autonomy slightly increases and the rest of the dimensions decline.

## 1. Introduction

Quality of life (QoL) has frequently been operationalized as an economic or health-related indicator, but this narrow measurement has been criticized [1]. What these authors argue, following several sociological authors (Anthony Giddens, Ulrich Beck, and Zygmunt Bauman), is that in current societies, at least in the so-called advanced ones, QoL is no longer determined by economic survival and/or health problems and diseases. These authors argue that ‘the contemporary phase of modernity (or postmodernity) is one where the personal is central and the construction of identity the ever-present task for everybody’ (p. 240) [1]. This person-centered and subjective approach is not new and was also held by the World Health Organization’s Quality of Life assessment group, which stated a widely accepted approach of QoL: ‘QoL assesses individuals’ perception of their position in life in the context of the culture and value systems in which they live and in relation to their goals, expectations, standards and concerns’ (p. 1403) [2].

That said, regarding QoL in the general population is even more obvious for the older population. Older adults’ QoL is of paramount interest for gerontologists, but it lacks a sufficiently agreed-upon definition, as well as theoretically grounded models for its measurement [3]. Due to the lack of theoretically grounded measures of QoL, indicators of health status have been used as proxies [4]. This has given rise to many instruments used in clinical and medical settings acknowledged as ‘health-related QoL’ [3]. Indeed, there are many authors who claim that the main problem for measuring QoL in old age is that it remains undertheorized and poorly defined [5,6,7].

One attempt to overcome this state of affairs was the development of the Control, Autonomy, Self-realization and Pleasure-19 (CASP-19) scale [3]. This scale is theoretically driven by the ‘needs satisfaction’ approach to measure QoL in early old age. It is based on Maslow’s Hierarchy of Needs [8]. This model includes four dimensions: Control, Autonomy, Self-realization and Pleasure. Following Wiggins et al. [9], Control and Autonomy are previous conditions to feel able to participate in society, while the extent to which these feelings can be realized is shown in the self-realization and pleasure dimensions. The dimension of pleasure is also extremely aligned with the theories on subjective well-being [10]. The CASP-19 is composed of 19 items tapping these four theoretical dimensions with four response categories. This version of the scale has been used in many local, regional, national and international studies [9,11]: the English Longitudinal Study of Ageing (ELSA); the British Household Panel Survey (BHPS); the Boyd-Orr survey; the Health, Alcohol and Psychosocial factors in Eastern Europe (HAPIEE) Study; the American Health and Retirement Survey; the Korean longitudinal Study of Ageing (KLoSA); the Irish Longitudinal Study of Ageing; the GAZEL Study; and the CONSTANCE study, among others.

Wiggins et al. [9] were the first ones to assess the factor structure and other psychometric properties of the CASP-19. The theoretical four-factor structure, either with a second-order factor or only first-order factors, did not achieve a good model fit, and a shortened 12-item version was proposed. These authors additionally proposed collapsing control and autonomy into a single factor. Since then, several studies have analyzed the psychometric properties of the CASP-19. Results of these validations suggest that the four-factor structure (either with or without a second-order factor) is compromised, while solutions collapsing control and autonomy, and self-realization and pleasure obtained better fit, while results also showed reliability of autonomy to be deficient [12,13,14,15]. Most of these studies included the shortened 12-item version and concluded that its psychometric characteristics were better [9,15].

Indeed, the version employed across the different waves of the Survey of Health, Ageing and Retirement in Europe (SHARE) is that composed of 12 items. This version of the instrument, the CASP-12, has also been validated in several studies. For example, Borrat-Besson et al. [16] analyzed all countries in SHARE Wave 4 and found that the theoretical four-factor structure of the CASP did not fit the data well. Instead, they proposed a two-factor structure (control/autonomy and self-realization/pleasure) and a further reduction to 10 items. Along the same lines, a study by Towers et al. [17] also found ill fit for the four theoretical domains and performed an Exploratory Factor Analysis (EFA) that found a three-dimensional structure: control, independence and global QoL, additionally deleting another item. Kerry [18], employing Item Response Theory (IRT) models, found a bifactor model with a strong global factor of QoL to better represent CASP-12 scores, with data from SHARE Wave 6.

Nevertheless, other studies have found good fit for the four-factor theoretical structure in the CASP-12. For example, Hamren et al. [19] found a good fit for the four-factor structure and good reliability in older Ethiopians, although they had to delete one item. Pérez-Rojo et al. [11] tested several Confirmatory Factor Analysis (CFA) models in Spanish-dwelling older adults (one, three and four first-order factors and a second-order factor model including four first-order factors). The best-fitting model had four first-order factors, but two items of autonomy had low loadings, and reliability of the autonomy dimension was poor. Finally, Rodríguez-Blázquez et al. [20] used Portuguese participants in the sixth wave of SHARE to test for the four-factor structure in CASP-12 scale and found good fit, again with low reliability estimates of the autonomy and pleasure dimensions.

In sum, setting the factor structure of a scale is critical in order to study its psychometric properties, and this structure has not been clearly established for the CASP-12. Since this scale is being widely used in a good number of international studies, the aim of this study is threefold: (a) to test the factor structure of the CASP-12 in the data from SHARE Wave 7; (b) to establish reliability and external validity of the dimensions found; and (c) to test for measurement invariance of three age groups (60–75 years old, 76–85 years old, and 86+ years old) since the original CASP scale was designed for ‘early’ older adults and not for the oldest-old.

## 2. Methods

### 2.1. Sample and Procedure

This study was carried out using data from the SHARE wave 7 [21,22]. SHARE is a longitudinal study focused on the study of European populations aged 50 and older. Data were gathered using probability-based sampling, further details of which can be found in Bergmann et al. [23].

The data included a total of 61,355 Europeans aged 60 years old or older from the 7th wave of SHARE (including Israel). Of the sample, 55.9% was female and the remaining 44.1% was male. The mean age was 71.87 years (Standard Deviation, SD = 8.23). Most were either living with their spouse (67.2%) or had become widowed (18.6%), while the rest had registered partnership (1.1%), lived separated from their spouse (1.1%), had never married (4.5%), or were divorced (7.4%). Mean years of education was 10.68 (SD = 4.28).

### 2.2. Instruments

The CASP-12 scale is a modification of the original CASP-19 [3]. The scale was designed to tap four dimensions of QoL: control, autonomy, self-realization, and pleasure. Answers are given in a Likert scale with four points, from ‘never ’ to ‘often’. Higher scores indicate a higher position on each dimension.

The European depression scale (EURO-D) [24] summarizes depression symptoms from various instruments on late-life depression used in different European countries. The scale comprises 12 items with dichotomously coded responses (absence vs. presence): depressed mood, pessimism, suicidal tendencies, guilt, sleep problems, loss of interest, irritability, loss of appetite, fatigue, concentration problems, enjoyment, and tearfulness. A scale score of 4 or higher could be considered as ‘case of depression’ and a scale score below 4 as ‘not depressed’ [25].

The self-perceived health measure rates present general health on a 5-point Likert scale between ‘excellent’ and ‘poor’. It is based on the 36-item Short-Form Health Survey (SF-36) [26] and uses the question ‘Would you say your health is…?’

Life satisfaction was measured with a single indicator asking about the respondents’ degree of satisfaction with their life, ranging from 1 (least satisfied) to 10 (most satisfied).

### 2.3. Statistical Analyses

SPSS 26 was used for calculating descriptive statistics of the variables under study, Cronbach’s alpha coefficients, corrected item-total correlations, and correlations among the dimensions in the CASP-12 and external criteria. Additionally, an R function [27] was used for alpha coefficients confidence intervals. The factor structure was tested using a series of competing Confirmatory Factor Analyses (CFA), estimated with Weighted Least Squares Mean and Variance corrected (WLSMV) in Mplus 8.4 [28]. This method of estimation was selected because the variables are ordinal and not multivariate normal [29,30]. Model fit was assessed with the most widely employed fit indexes. Specifically, we used the chi-square statistic; the Comparative Fit Index (CFI); the Root Mean Square Error of Approximation (RMSEA), with a 90% Confidence Interval (CI); and the Standardized Root Mean Square Residual (SRMR). The adopted criteria for accepting a model were those in Hu and Bentler [31] and Marsh et al. [32]: a CFI of at least 0.90, together with a RMSEA and SRMR less than 0.08, indicate adequate fit, while a CFI of at least 0.95 and RMSEA and SRMR below 0.08 indicate excellent fit. The Composite Reliability Index (CRI) for each of the scale’s dimensions was calculated using standardized factor loadings in the best-fitting CFA.

Finally, a standard measurement invariance routine was estimated including the testing of three CFAs: configural invariance model, weak or metric invariance model, and strong or scalar invariance model [33]. The configural model estimates the four-factor model in the three age groups at the same time, with separate estimates for each group. The fit indexes of this configural model are used as the baseline fit. The metric or weak invariance model sets factor loadings to be equal across groups. The scalar or strong invariance model further constraints items’ thresholds in the intercepts to equality. The models in this sequence are nested, and therefore they may be compared with chi-square differences (in the case of WLSMV estimation, the DIFFTEST). Non-significant chi-square differences suggest multi-group equivalence or invariance. However, this test is extremely powerful in detecting trivial differences, especially with relatively large samples [34,35]. Therefore, a modeling approach has been advocated which employs CFI differences <0.01 as cut-off criteria to accept the more parsimonious model [34]. If a more parsimonious model evinces adequate levels of practical fit, then the imposed constraints are considered a reasonable approximation for modeling the data, and invariance at that level is declared.

## 3. Results

### 3.1. Factor Structure

Several competing CFAs were estimated. These competing models come from the structures that were supported in previous validations of the CASP-12. Specifically, the CFAs tested were:

(1) One-factor model, found in Kerry [18].
(2) Two-factor model (control/autonomy and self-realization/pleasure), supported, for example, by Borrat-Besson et al. [16].
(3) Three-factor model (control/autonomy, self-realization, and pleasure), found, for example, in Stoner et al. [15].
(4) Four-factor model (control, autonomy, self-realization, and pleasure), theoretically proposed during the scale development.
(5) Four-factor model with a second-order factor (QoL), also based on the theory underlying the scale development.

The goodness-of-fit indexes for all tested models are presented in Table 1. The best-fitting model is that originally thought for the scale. That is, the four correlated factors model. Nevertheless, the fit of the second-order factor model is also very good, and extremely similar to the fit of the four correlated factors model. Given that the second-order model is more parsimonious and opens the possibility of using the scale with the dimensions or as a general factor, depending on research interests, this second-order model will be retained.

**Figure 1:**
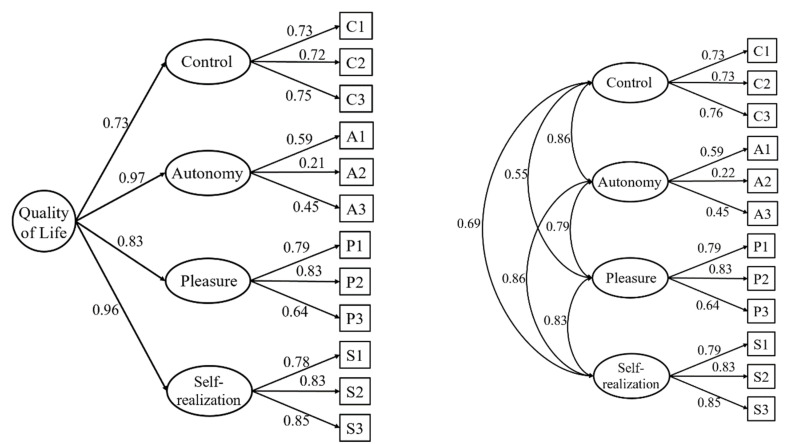
Standardized factor loadings for the retained model. Note: all estimates p < 0.01.

Standardized parameter estimates are presented in Figure 1. Although all the factor loadings, both in the first-order factors and the second-order factor, were statistically significant (*p* < 0.01), the second item in the autonomy dimension had a relatively low factor loading in this dimension. This item has repeatedly been found to be problematic in the literature. Its content is ‘Family responsibilities prevent me from doing what I want to do’.

### 3.2. Internal Consistency

For the overall scale and the four first-order factors, both the alpha coefficient and the CRI were estimated. The alpha for the global measure of QoL was 0.833 (95% CI (0.831–0.834)), and the CRI was also very high (CRI = 0.932). Regarding the reliabilities of the four dimensions, all of them were adequate except the estimates of autonomy. In the case of control, the alpha was 0.709 (95% CI (0.705–0.712)) with a CRI = 0.784. Self-realization had an alpha of 0.816 (95% CI (0.813–0.818)) and a CRI of 0.865. Pleasure’s estimates of reliability were adequate: alpha = 0.695 (95% CI (0.691–0.698)), and CRI = 0.801. The two estimates of reliability, alpha and CRI, were inadequate for the autonomy dimension: alpha = 0.351 (95% CI (0.342–0.359)), and CRI = 0.394.

Table 2 presents basic descriptive statistics for the 12 items in the scale, means and standard deviations. It also shows the corrected item-total correlations.

### 3.3. External Validity

The correlation coefficients are displayed in Table 3, with all being statistically significant (*p* < 0.001). As a global score for QoL, CASP-12 is positively and similarly highly related to satisfaction with life and self-perceived health, and negatively correlated with depression. The highest positive association with CASP-12 dimensions is found with life satisfaction. As expected, all CASP dimensions negatively correlate with depression, as measured by EURO-D, with the pleasure dimension (−0.498, *p* < 0.001) being the one with the strongest association.

### 3.4. Measurement Invariance

Given that the original scale (CASP-19) was developed to be used in early old age, and first validated for older adults in the age range of 65 to 75 years, we consider it important to test for measurement invariance outside this age range. Therefore, a measurement invariance routine across age groups was tested. Age was divided into three groups: 60–75, or early old adults; 76–85, or old adults; and 86+, or oldest-old.

Goodness-of-fit indexes and chi-square differences for the invariance routine are shown in Table 1. DIFFTESTS (chi-square differences) were all statistically significant, but as already mentioned, due to the power of this test, that was expected. Therefore, CFI differences will be used to establish invariance. The configural model fitted the data well and established the baseline fit. When factor loadings were constrained to be equal across groups (metric invariance), model fit improved, and therefore the CASP-12 could be considered to be metric invariant. Furthermore, when all thresholds were fixed to be equal across groups (scalar invariance), fit slightly worsened (CFI difference = 0.014). Attending to Modification Indexes, one constraint, threshold for item 4, was released, and a modified scalar-invariant model was tested. This model again reached very good fit with no statistically significant differences. Therefore, except for item 4’s threshold, the CASP-12 can also be considered scalar invariant.

Once scalar invariance was established, latent mean differences could be calculated. Table 4 offers these latent differences, their statistical significance, and Cohen’s *d* as an estimator of the effect size. The reference group is the age group 60–75. As can be seen in Table 4, all differences between the reference group and group 2 (76–85) and group 3 (86+) were statistically significant, with effect sizes being small to moderate. The pattern of differences is clear. As people age, control, self-realization and pleasure decline, while autonomy slightly increases.

## 4. Discussion

It is nowadays accepted that measuring the extent to which needs are fulfilled provides a measure of QoL richer than an overall personal assessment (such as life satisfaction), allowing for comparisons between people’s different QoL scores [1]. Once the indubitable interest of approaching QoL’s measurement from a contemporary, more sociological, perspective is established, a measure such as the CASP-12 produces more interest. This measure includes eudaimonic and hedonic components, and it has been widely used in international studies [9,11]. In particular, it has been selected as the measure of QoL for protocols in different international longitudinal surveys, and the evidence on its psychometric properties points out that it outperforms the CASP-19 [9,15].

Despite its extended use and its clear four-dimension conceptualization, its factor structure has been controversial since the beginning; different studies in different populations have found different structures [16,17,18]. Therefore, analyzing the large and new database of the SHARE longitudinal study may aid in shedding light onto the factor structure of the CASP-12, at least in older populations.

For the sake of completeness, all factor structures with empirical (and/or theoretical) support in the literature have been tested: one-factor model [18]; two-factor model based on control/autonomy and self-realization/pleasure [16]; three-factor model with control/autonomy, self-realization, and pleasure [15]; four-factor model (control, autonomy, self-realization, and pleasure) as theoretically proposed during the scale development; and finally a four-factor model with a second-order factor (QoL). According to the underlying theory, control and autonomy are two domains that constitute prerequisites for being able to participate in society, and the extent to which these feelings of freedom are fulfilled is captured by the self-realization and pleasure dimensions [9].

Regarding the best-fitting factor structure, a second-order solution with four subscales was retained. On the one hand, the studies by Stoner et al. [15] and Wiggins et al. [9] both found this solution to better represent the data, even when compared to the 19-item version. On the other hand, among those studies contemplating the 12-item version only, a variety of solutions have been suggested: one-factor model of QoL [18], two-factor model of control/autonomy and self-realization/pleasure [16], three-factor model of control independence and global QoL [17], and four-factor model of control, autonomy, self-realization and pleasure [11,19,20]. However, work by Borrat-Besson et al. [16] and Towers et al. [17] recommended deletion of two and one items, respectively. This was also the case for Hamren et al. [19]. For their part, Pérez-Rojo et al.’s [11] results suggested that both four first- and second-order factor solutions fitted the data well. Nevertheless, in these models, item 5 and item 6 presented low factor loadings: 0.35 and 0.35 in the autonomy dimension of both models. Rodríguez-Blázquez et al. [20] also found diminished factor loadings of items 5 (0.25) and 6 (0.31) in the autonomy dimension. Our study found a similar low loading of item 5 (referred to as A2 in Figure 1, 0.22) and a moderate factor loading of item 6 (referred to as A3 in Figure 1, 0.45).

Reliability for the CASP-12 in this research was adequate for the overall scale as well as for control, self-realization and pleasure, but the autonomy dimension’s alpha and CRI were quite low. These results are similar to other studies. For the overall CASP-12, reliability measured by alpha was 0.83, ranging from 0.35 (autonomy) to 0.82 (self-realization) for the domains, very similar to Rodríguez-Blázquez et al. [20], who reported values for overall CASP-12 of 0.78 with values between 0.37 (autonomy) and 0.73 (self-realization) for the dimensions. This same pattern of findings was found by Pérez-Rojo et al. [11], with an alpha value of 0.86 for the overall scale, and alpha estimates between 0.39 and 0.82 for the four dimensions. Across all revised studies, the reliability of the autonomy domain was the weakest one [9,16,36]. The autonomy domain refers to self-determination and the absence of unwanted interference from others. It is an inner endorsement of one’s actions, the sense that they emanate from oneself and are one’s own [37]. In our study (and others), problems of reliability within this dimension came from its first item (‘I can do the things that I want to do’).

Given that the CASP was originally designed for ‘early’ older adults, the age of the samples could contribute to the differences in factor structure obtained so far. Previous research carried out with the CASP-12 SHARE version has included people aged 50 and older and sometimes included a small proportion of people below 50 years old [16]. Therefore, a relevant contribution of this work is the formal test of measurement invariance across age groups (60–75 years old, 76–85 years old, and 86+ years old). Results showed that CASP-12 could be considered scalar invariant only with the exception of one threshold for item 4 (‘I can do the things that you want to do’). Baltes and Smith [38] pointed out the important distinction between the third and fourth ages, the latter being what is commonly referred to as the ‘oldest-old’. This age bracket is gaining more and more attention, which should come as no surprise, given that it is growing at a proportionately higher rate than the younger brackets. In fact, the proportion of people aged 80 or over based on United Nations Population Division [39] is growing twice as quickly as the 60 and older bracket.

Generally speaking, population studies in developed countries show that measures of wellbeing (such as life satisfaction) remain steady throughout life [38], perhaps with minor fluctuations [40]. However, the ‘oldest-old’ (people over the age of 80) do seem to notice a pronounced decline in life satisfaction [41,42,43]. Anyway, many studies with old people have found a slight drop in life satisfaction with age [44,45].

Previous CASP-12 validation studies used samples with a wide range of age [17,18,20] not paying attention to potential age group differences, both in structure and mean levels. Regarding factor structure, this research points out that the scale is psychometrically sound across ages. Regarding mean differences, we found evidence that QoL decreases over time, and this decrease is greater in the oldest-old [46,47,48]. In a longitudinal study using CASP-12 and latent growth models, Ward et al. [49] found that QoL decreased non-linearly with age. However, this was not true for autonomy. We have no clear explanation for this result, but we may anticipate some tentative reasons. First, this is the dimension with the lowest reliability. Second, two items of autonomy are very specific about why autonomy is lacking: because of family responsibilities and/or shortage of money. These difficulties for autonomy may be present at different times in life, but less present in very old age.

When compared to previous evidence on external validity, the correlations between the CASP-12 and the EURO-D (depression) supported previous research on the negative relation between QoL and depression [50,51]. Additionally, Portellano-Ortiz et al. [52], using data from SHARE Wave 5, found moderate or strong relations with depression (r = −0.59) in all European countries, and, albeit using an indicator of physical rather than self-perceived health (very good, good, fair, poor), similar results were found for the association between QoL and health (0.51 vs. 0.48). Similarly, CASP-12 total scores from the Portuguese sample of the 6th wave of SHARE correlated −0.57 with depression, 0.52 with life satisfaction and similar correlation (0.47) with self-perceived health [20]. For the domains, while in the 6th wave in Portugal, the pleasure factor showed the lowest correlation with the external variables; the current research using wave 7 and including all Europeans showed the autonomy dimension to have the lowest one. These differences could be affected by the exceptionally low reliability found in Portugal for the pleasure dimension (α = 0.34) in wave 6 [14].

All in all, this study provides evidence of CASP-12’s construct validity for data coming from the 7th wave of SHARE, while also acknowledging some issues related to the autonomy dimension, such as low factor loadings which simultaneously lead to diminished estimated reliability. This study also fills the gap in the literature regarding CASP-12’s adequacy for use in oldest-old adults, given that it was initially designed for ‘early’” older adults. Future research should aim to study the scale’s psychometric characteristics across regions, as QoL may be culture-dependent.

## Figures and Tables

**Table 1:** Goodness-of-fit indexes for the five CFAs and the measurement invariance routine.

Model	χ^2^	df	*p*	RMSEA	90%CI	CFI	SRMR	Δχ^2^	df	*p*	ΔCFI
One-factor model	48,906.4	54	<0.001	0.123	0.123–0.124	0.903	0.062	-	-	-	-
Two-factor model	28,867.4	53	<0.001	0.096	0.095–0.097	0.943	0.048	-	-	-	-
Three-factor model	21,564.1	51	<0.001	0.084	0.083–0.085	0.957	0.044	-	-	-	-
Four-factor model	16,443.1	48	<0.001	0.076	0.075–0.077	0.968	0.038	-	-	-	-
Second-order model	19,963.1	50	<0.001	0.082	0.081–0.083	0.961	0.043	-	-	-	-
*Measurement invariance*											
Configural	14,656.3	144	<0.001	0.071	0.070–0.072	0.969	0.037	-	-	-	-
Metric	13,461.7	160	<0.001	0.065	0.064–0.066	0.972	0.038	887.2	16	<0.001	0.003
Scalar	19,973.2	200	<0.001	0.071	0.070–0.072	0.958	0.041	7233.4	40	<0.001	0.014
Modified scalar	17,266.5	198	<0.001	0.066	0.065–0.067	0.964	0.040	4607.9	38	<0.001	0.008

Note. CFA= Confirmatory Factor Analyses; χ^2^ = chi-square statistic; df = degrees of freedom; p = probability; RMSEA = Root Mean Square Error of Approximation; 90%CI = 90% Confidence Interval; CFI = Comparative Fit Index; SRMR = Standardized Root Mean Square Residual; Δχ^2^ = differences in chi-square; ΔCFI = differences in Comparative Fit Indexes.

**Table 2:** Means, standard deviations (SD), and corrected item-total correlations (**r_it_**) for the 12 items in the CASP measure (Control, Autonomy, Self-realization and Pleasure).

Item	Mean	SD	r_it_
Control 1	2.53	1.04	0.48
Control 2	2.79	0.99	0.58
Control 3	3.14	0.95	0.52
Autonomy 1	3.18	0.89	0.13
Autonomy 2	3.09	0.96	0.20
Autonomy 3	2.61	1.10	0.28
Pleasure 1	3.47	0.77	0.29
Pleasure 2	3.47	0.79	0.32
Pleasure 3	3.37	0.76	0.20
Self-realization 1	3.06	0.87	0.39
Self-realization 2	3.03	0.89	0.48
Self-realization 3	2.98	0.91	0.48

**Table 3:** Correlations among CASP dimensions, CASP-12 and criteria (all statistically significant *p* < 0.001).

	(1)	(2)	(3)	(4)	(5)	(6)	(7)
(1) Control	1						
(2) Autonomy	0.442	1					
(3) Self-realization	0.365	0.334	1				
(4) Pleasure	0.528	0.391	0.609	1			
(5) CASP-12	0.789	0.694	0.732	0.836	1		
(6) Life satisfaction	0.406	0.366	0.472	0.510	0.573	1	
(7) Depression	−0.442	−0.257	−0.422	−0.498	−0.524	−0.416	1
(8) Self-perceived health	0.405	0.233	0.304	0.494	0.479	0.361	−0.439

**Table 4:** Latent mean differences and effect size estimators.

	Group 2 vs. 1			Group 3 vs. 1		
Factor	$\bar{X}$ Difference	*p*	Cohen’s d	$\bar{X}$ Difference	*p*	Cohen’s d
Control	−0.514	<0.001	0.471	−0.950	<0.001	0.811
Autonomy	0.196	<0.001	0.234	0.668	<0.001	0.655
Self-realization	−0.628	<0.001	0.498	−1.111	<0.001	0.478
Pleasure	−0.365	<0.001	0.290	−0.598	<0.001	0.857

## Data Availability

The SHARE data are distributed by SHARE-ERIC (Survey of Health, Ageing and Retirement in Europe—European Research Infrastructure Consortium) to registered users through the SHARE Research Data Center. The SHARE Research Data Center (FDZ-SHARE) complies with the Criteria of the German Council for Social and Economic Data for providing access to microdata. Börsch-Supan, A. (2020). Survey of Health, Ageing and Retirement in Europe (SHARE) Wave 7. Release version: 7.1.1. SHARE-ERIC. Data set. DOI: 10.6103/SHARE.w7.711.
